# Opposing responses of the rat pulmonary artery and vein to phenylephrine and other agents in vitro

**DOI:** 10.1186/s12890-021-01558-8

**Published:** 2021-06-05

**Authors:** Li-mei Liao, Li Zhou, Chen-ran Wang, Jian-ying Hu, Yao-jun Lu, Shaoqiang Huang

**Affiliations:** 1 Department of Anaesthesia, Obstetrics and Gynecology Hospital, Fudan University, 128 Shenyang road, Shanghai, 200090 China; 2 Department of Physiology and Pathophysiology, School of Basic Medicine Science, Fudan University, 130 Dongan Road, Shanghai, 200032 China; 3 Department of Anesthesiology and Perioperative Medicine, Guangzhou Women and Children’s Medical Center, Guangzhou Medical University, 9# Jinsui Road, Guangzhou, 510623 China

**Keywords:** Arginine vasopressin, Phenylephrine, Potassium chloride, Pulmonary artery, Pulmonary vein, Vascular tension

## Abstract

**Background:**

Different from current cognition, our study demonstrated that adrenergic receptors agonist phenylephrine significantly relaxed isolated pulmonary artery but constricted pulmonary veins. Through comparing differences in the effects of commonly used vasoactive drugs on pulmonary artery and veins, the study aimed to improve efficiency and accuracy of isolated pulmonary vascular experiments, and to provide experimental basis for clinical drug use.

**Methods:**

The contractile responses of pulmonary arteries and veins from twelve-week-old Male Sprague-Dawley rats to phenylephrine, arginine vasopressin (AVP), U46619, endothelin-1, and potassium chloride (KCl) were recorded, as well as the relaxation in response to phenylephrine, AVP, acetylcholine. To further explore the mechanism, some vessels was also pre-incubated with adrenergic receptors antagonists propranolol, prazosin and nitric oxide synthesis inhibitor N[gamma]-nitro-L-arginine methyl ester (L-NAME) before addition of the experimental drugs.

**Results:**

Phenylephrine constricted pulmonary veins directly, but constricted pulmonary artery only after incubation with propranolol or/and L-NAME. The pulmonary artery exhibited significant relaxation to AVP with or without L-NAME incubation. AVP more clearly constricted the veins after incubation with L-NAME. Changes in vascular tension also varied from pulmonary artery to veins for KCl stimulation. Different from phenomena presented in veins, acetylcholine did not relax pulmonary artery preconstricted by KCl, U46619, and endothelin-1.

**Conclusions:**

According to the results, phenylephrine, KCl, AVP, and acetylcholine could be used to distinguish pulmonary arteries and pulmonary veins in vitro. This also suggested that the pulmonary arteries and pulmonary veins have great differences in physiology and drug reactivity.

## Background

Phenylephrine, a sympathomimetic amine, was previously believed to possess a pure α-adrenergic receptor agonist with little β-adrenergic receptor activity. It induces arteriolar vasoconstriction to increase systemic vascular resistance and mean blood pressure. Phenylephrine, an instrumental drug, is also widely used in in vivo and ex vivo studies investigating the contraction of blood vessels, and in combination with acetylcholine to assess the integrity of the vascular endothelium.

The pulmonary artery is the resistance vessel of the right ventricular outflow tract. It is commonly used in vascular tension experiments investigating the pathogenesis and treatment of pulmonary hypertension. Presently, researchers generally believe that phenylephrine, as a vasoconstrictor drug, exerts a contractile effect in the pulmonary artery. However, previous in vitro studies have reported a significant variety of force generation in response to phenylephrine in pulmonary artery [1–6]. In 1999, a study by Lal et al. demonstrated that rat pulmonary veins under normal control and those in a chronic hypoxia model exhibited little contraction after stimulation by a phenylephrine concentration gradient, while the pulmonary artery exhibited concentration-dependent contraction [7]. In our experiments, however, an interesting phenomenon was observed. Stimulation of an isolated rat pulmonary artery using phenylephrine did not produce a contractile response, but significantly increased the vascular tone of the pulmonary vein. Based on inconsistent results reported in the literature, we questioned the differences in the contractile effects of phenylephrine on the pulmonary artery and veins, and the potential effector mechanisms.

Recent study have confirmed that phenylephrine can inhibit isolated myometrium contraction in mice by activating the β_2_ receptor [8]. In in vivo experiments, it has also been reported that endogenously released epinephrine attenuates pulmonary vasoconstriction and bronchoconstriction in systemic allergic reactions in Sprague-Dawley rats by activating β_2_-adrenergic receptors [9]. As such, does phenylephrine also inhibit pulmonary artery contraction and even produce a relaxation effect by activating β-receptors? Moreover, what role does nitric oxide (NO), the active component of endothelium-derived relaxing factor (EDRF), play? At the same time, our experiments revealed that phenylephrine clearly resulted in contraction of the pulmonary veins, suggesting that there is a significant difference in vascular reactivity to phenylephrine between the pulmonary arteries and veins. Furthermore, do other vasoactive drugs, which are commonly used in experiments, still have similar opposite phenomena on pulmonary arteries and veins.

Rodents are often used to study the pathophysiology of lung diseases. Based on the above questions, the present research aimed to explore the difference(s) in the vascular reactivity of healthy rat isolated pulmonary arteries and veins to phenylephrine, and to explore its mechanism of action. In addition, differences in the effect of arginine vasopressin (AVP), endothelin, U46619, and other common vasoactive drugs on the pulmonary artery and pulmonary veins were observed. Exploring these issues would be beneficial in distinguishing intralobar pulmonary arteries and veins, which could improve the efficiency and accuracy of isolated pulmonary vascular experiments and have more clear knowledge to pulmonary vessels, and to provide an experimental basis for clinical drug use.

## Methods

### Animal preparation

Approved by the Animal Ethics Committee of Shanghai Medical College of Fudan University [Approval number: 201907008Z], twelve-week-old Male Sprague-Dawley rats (343 ± 11 g), purchased from Shanghai Jiesijie Experimental Animal Co. LTD., were housed for 4 days in the animal facility and maintained on ad libitum standard rat chow and tap water in 12:12-h light-dark cycle. In the present experiment we total used 37 rats. The number of rats used in different experimental parts had been indicated in the corresponding legends. In order to reduce unnecessary sacrifices, datas from some rats were reused (N = 14 in Fig. 5). Rats were administered pentobarbital via intraperitoneal injection at 140 mg/kg for euthanasia. After breathing and heartbeat disappear, the chest was quickly opened, and the heart and lungs were removed and immersed in cool, oxygenated physiologic salt solution (PSS) with the following composition: (mmol)NaCl, 118.3 ; KCl,4.7 ; MgSO_4_,1.2 ; KH_2_PO_4_, 1.22; CaCl_2_, 2.5; NaHCO_3_, 25.0; calcium-ethylenediaminetetraacetic acid, 0.016, and glucose, 11.1 .

### Isolation of pulmonary artery and veins

The superior and inferior vena cava were used as landmarks to confirm the location of the right atrium, and to search for the pulmonary artery at the hilum, upward from the right atrium. The pulmonary vein was searched upward from the left atrium. After fully triming lung parenchyma that sticked to pulmonary vascular, the pulmonary vein segments with internal diameter ~ 200 μm and the pulmonary artery segments with internal diameter ~ 400 μm, all in 3 mm in length, were transected from the lung.

### In vitro experiments

Segments of the pulmonary blood vessels were suspended in organ chambers filled with control solution (5 ml) maintained at 37 °C and bubbled with 95 % oxygen and 5 % carbon dioxide (pH 7.4) in a vascular tone measurement system (620 M, Danish Myo Technology, Denmark). Each blood vessels was equilibrated for 1 h at the mean optimal resting tension (5 mN). After equilibration, 60 mM potassium chloride (KCl) was added to fully activate the vascular ring and evaluate vascular activity. In the present experiments, the vascular endothelium was divided into nondisruptive endothelium and disrupted endothelium, the later was damaged by gently rubbing the intimal surface of the vessel using a pair of watchmaker’s forceps [10]. In the present experiment, the pulmonary vessels were randomly assigned to different treatment groups.

In contraction experiments, endothelin-1 (10 pM to 0.1 µM), phenylephrine (1 nM to 100 µM), or AVP (10 pM to 1 µM) were added in cumulative half-log_10_ increments, allowing time for the response to plateau between additions. Contractions in response to each drug concentration are expressed as %KPSS maximum contraction within the blood vessels.

In experiments investigating relaxation of the pulmonary artery in response to phenylephrine or AVP, the pulmonary arteries were precontracted to 60–75 % KPSS maximum using U46619 (a thromboxane A2 receptor agonist [300 to 800 nM]) [7, 10]. AVP and phenylephrine were added in cumulative half-log_10_ increments allowing time (approximately 2 min) for the responses to plateau between additions. Control experiments were also performed to determine the effect of time on U46619-induced tone. In some parts of the study, the vascular ring was pre-incubated with receptor antagonists (5 × 10^− 6^ M propranolol and 2 × 10^− 5^ M prazosin) or inhibitors (2 × 10^− 4^ M N[gamma]-nitro-L-arginine methyl ester [L-NAME]) for 30 min before addition of the experimental drug(s) [7, 11]. And in the present study all control group was added solvent distilled water or dimethyl sulfoxide the same as corresponding experiment group.

Vascular rings that did not respond to KCl were excluded from further experimentation. And blood vessels that disconnected from the pulmonary arteries or veins trunk before removing of the lung connective tissue around the target vessels would also not be used in the experiment, which aimed to ensure that the isolated blood vessel was the exact pulmonary artery or pulmonary vein.

### Drugs

The drugs used in the experiments were all purchased from MedChemExpress: phenylephrine, acetylcholine, AVP, propranolol hydrochloride, prazosin hydrochloride, endothelin-1 and L-NAME (Calbiochem, San Diego, CA, USA). All powdered drugs were prepared using distilled water, except for propranolol and prazosin, which were dissolved in dimethyl sulfoxide. The concentrations are expressed as final molar concentration in the organ chambers.

### Statistical analysis

All data are expressed as mean ± 1 standard error of the mean (SEM). Concentration-response curves were fitted using Prism version 8 software (GraphPad Inc, San Diego, Ca, USA) for each individual experiment. The differences between the groups at each concentration was analysed with one-way analysis of variance (ANOVA) and Bonferroni post hoc comparison (2-sided), or Student t-test, using SPSS version 22 (IBM Corporation, Armonk, NY, USA). In all experiments, N represented the number of animals from which blood vessels were obtained. In all cases, *P* < 0.05 was accepted as statistically significant.

## Results

### Contraction responses of the pulmonary artery and veins to phenylephrine

**Fig. 1:**
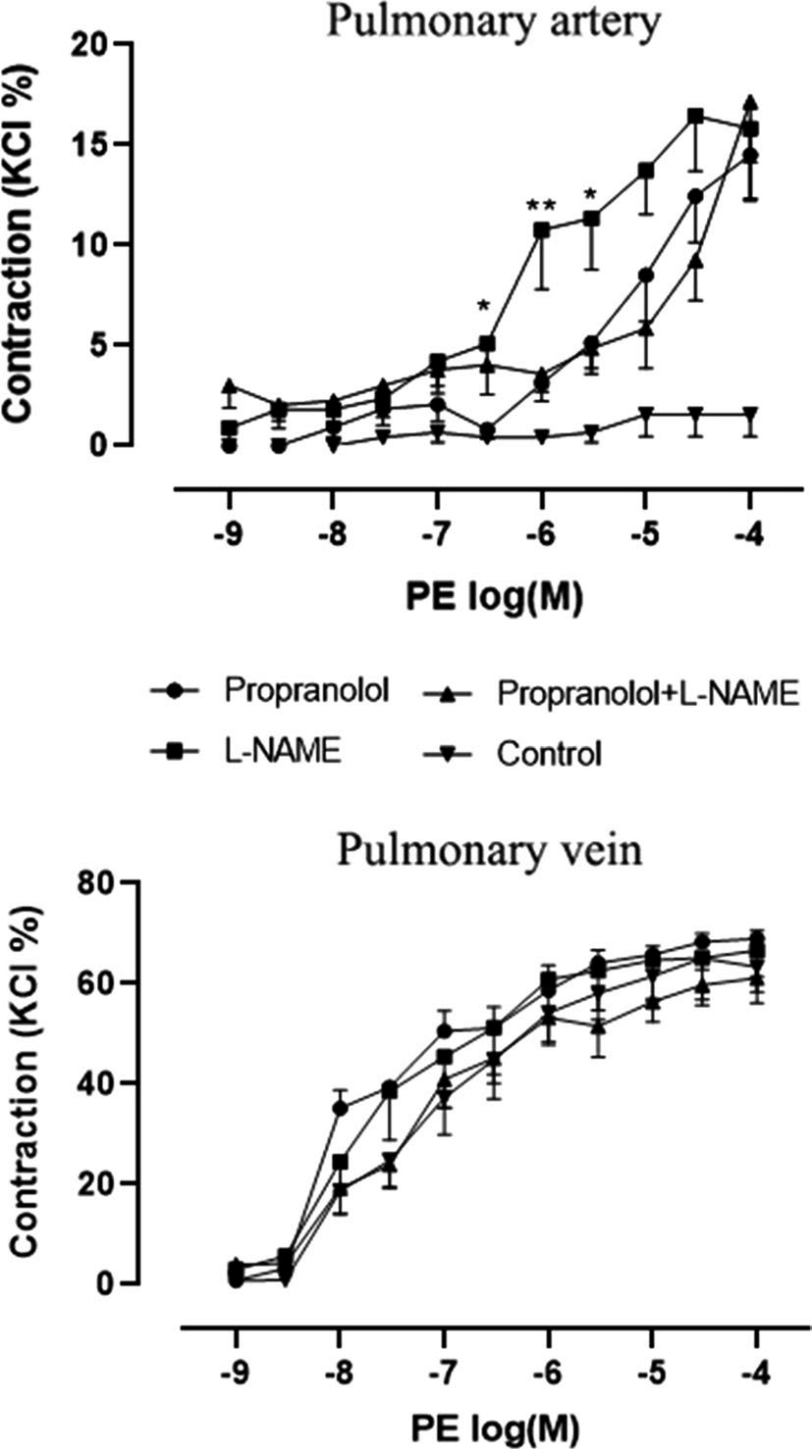
Constriction responses of the pulmonary arteries and veins to phenylephrine. Concentration-response curves to phenylephrine in segments of rats (N = 6–8) pulmonary arteries and pulmonary veins. The number of rats is the number of repetitions of each group. After pretreatment with propranolol or N[gamma]-nitro-L-arginine methyl ester (L-NAME) or both, arteries and veins were exposed to increasing concentrations of phenylephrine. Control group directly exposed to phenylephrine. Asterisk indicates artery segments pretreated with L-NAME were significance than segments pretreated with propranolol and combine propranolol and L-NAME. There were no significant difference in pulmonary arteries tension between pretreated with propranolol and combine propranolol and L-NAME. There were no significant difference in pulmonary veins tension among all groups. Where no error bar is visible the SEM is within the symbol. “*” means *p* < 0.05; “**” means *p* < 0.01. (one-way analysis of variance, Bonferroni post hoc comparison)

Phenylephrine itself did not constrict pulmonary artery. However, phenylephrine increased vascular tone in a concentration-dependent manner when the pulmonary arteries were pretreated with propranolol or L-NAME, or both, for 30 min. At 3 × 10^− 7^ M of phenylephrine, the difference only existed between the L-NAME pretreatment group and the propranolol pretreatment group. At 10^− 6^ M and 3 × 10^− 6^ M of phenylephrine, pulmonary artery tension after L-NAME pretreatment was significantly greater than that in pulmonary arteries pretreated with propranolol, and combined L-NAME and propranolol. There was no difference between the propranolol pretreatment group and the combined propranolol and L-NAME pretreatment group at each phenylephrine concentration. With increases in phenylephrine concentration, vascular tension in the L-NAME pretreatment group first increased and then decreased, while it did not decrease in the other two groups. However, the maximum vascular tension stimulated with phenylephrine did not exhibit a significant difference among the three groups (propranolol, 14.5 ± 2.3 %; L-NAME, 16.6 ± 1.9 %; combined, 17.2 ± 3.0 %; *p* = 0.721) (Fig. 1).

There was no difference in the contractile effect of phenylephrine on the pulmonary veins in each group with or without pre-incubation with propranolol or/and L-NAME (control, 67.3 ± 1.8 %; propranolol: 69.9 ± 1.7 %; L-NAME, 67.1 ± 8.2 %; combined [i.e., L-NAME and propranolol], 62.3 ± 4.5 %; *p* = 0.455) (Fig. 1).

### Relaxation response of pulmonary arteries to phenylephrine

**Fig. 2:**
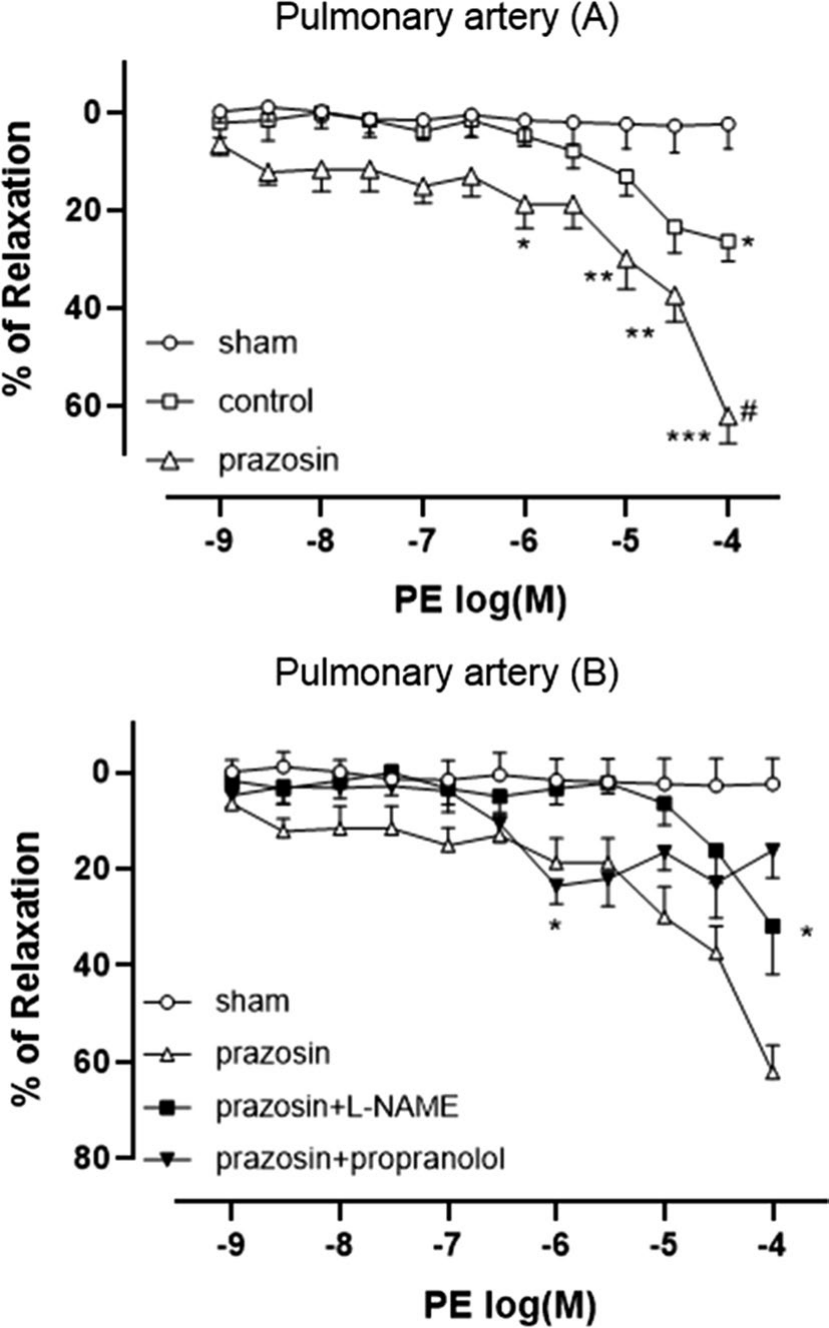
Relaxation responses of pulmonary arteries to phenylephrine. Concentration-response curves to phenylephrine in segments of pulmonary arteries (**a** and **b**) from rats (N = 5–7). The number of rats is the number of repetitions of each group. After pretreatment with receptor antagonists (5 × 10 ^− 6^ M propranolol and 2 × 10 ^− 5^ M prazosin) or inhibitors (2 × 10 ^− 4^ M N[gamma]-nitro-L-arginine methyl ester [L-NAME]) for 30 min, constriction of all arteries was induced with U46619, arteries then were exposed to increasing concentrations of phenylephrine except sham group. Control group was exposed to phenylephrine without any pretreatment. Asterisk indicates significance compared to the sham group. Hashtag indicates significance compared to the control group. “*” means *p* < 0.05; “**” means *p* < 0.01; “***” means *p* < 0.001; “#” means *p* < 0.001. (one-way analysis of variance, Bonferroni post hoc comparison)

After pretreatment with U46619 (sham group), the pulmonary artery tension did not decrease significantly. Phenylephrine reduced pulmonary artery tension in a concentration-dependent manner at drug concentrations > 10^− 6^ M, and was statistically significant at 10^− 4^ M. After 30 min of pre-incubation with prazosin, an α1 adrenergic blocker, the relaxation effect of phenylephrine was more distinct; comparing with the sham group, statistically significant differences were found at ≥ 10^− 6^ M, and at 10^− 4^ M compared with the control group (Fig. 2A).

Compared with the group incubated only with prazosin, the group preincubated with prazosin and L-NAME, the relaxation effect of phenylephrine was weakened at all concentrations, but was still significantly different at 10^− 4^ M. After 30 min-preincubation with prazosin and propranolol, significant relaxation effect of phenylephrine was produced only at 10^− 6^ M. Compared with the group incubated only with prazosin, the group preincubated with prazosin and propranolol almost completely reversed the relaxation effect when the phenylephrine concentration was > 10^− 6^ M (Fig. 2B).

### Constriction response of the pulmonary artery and veins to AVP

**Fig. 3:**
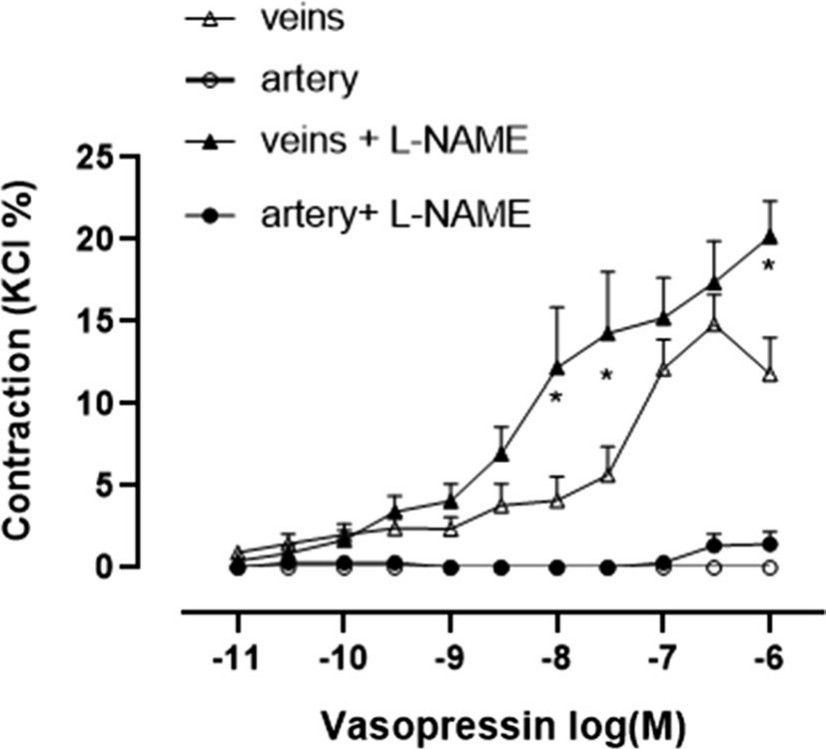
Constriction responses of the pulmonary arteries and veins to AVP. Concentration-response curves to AVP in segments of rats (N = 6) pulmonary arteries or veins. The number of rats is the number of repetitions of each group. Asterisk indicates significance of pulmonary vein segments between pretreated and unpretreated with L-NAME (*p* < 0.05, Student t-test)

Compared with pulmonary veins that were not incubated with L-NAME for 30 min, the pulmonary veins incubated with L-NAME exhibited an increased sensitivity to gradient concentrations of AVP, and vascular tone continued to increase with increasing AVP concentration, and did not decline or decay. Pulmonary artery did not response to AVP (Fig. 3).

### Relaxation response of the pulmonary artery to AVP

**Fig. 4:**
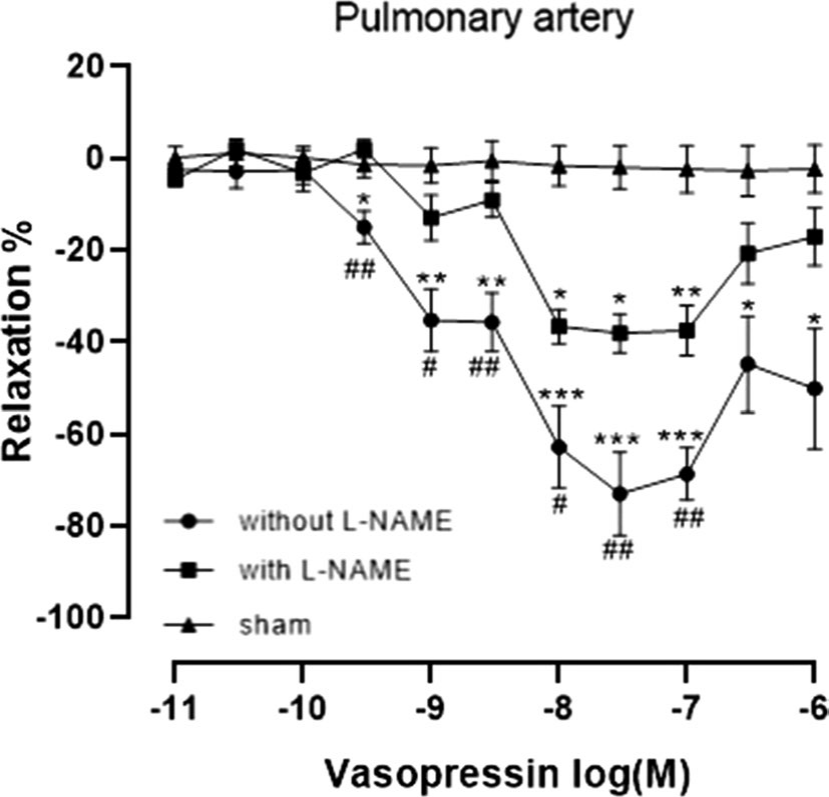
Relaxation responses of the pulmonary arteries to AVP. Concentration-response curves to AVP in segments of pulmonary arteries from rats (N = 5–6). The number of rats is the number of repetitions of each group. After the presence or absence of the L-NAME pretreatment, contraction was all induced with U46619, the segments were exposed to increasing concentrations of AVP except sham group. Asterisk indicates significance compared to the sham group. “*” means *p* < 0.05; “**” means *p* < 0.01; “***” means *p* < 0.001; The hashtag indicates the significance between the presence and absence of the L-NAME pretreatment. “#” means *p* < 0.05; “##” means *p* < 0.01 (one-way analysis of variance, Bonferroni post hoc comparison)

Comparing with sham group, pulmonary artery tension was significant lower at AVP concentrations ≧ 3 × 10^− 10^ M in the group without L-NAME incubation while only significant lower at 10^− 8^ to10^− 7^ M in group with L-NAME incubation. Regardless of whether pre-incubated with L-NAME, after pulmonary artery contraction induced by U46619, vascular tension first decreased and then increased with increases in AVP concentration. Maximal relaxation occurred at an AVP concentration of 3 × 10^− 8^ M in both AVP treatment groups. Compared with the pulmonary artery incubated with L-NAME for 30 min, the pulmonary artery without L-NAME incubation demonstrated more pronounced relaxation with increases in AVP concentration. Furthermore, vascular tension in the sham group did not decrease significantly during the experiment (Fig. 4).

### Response of pulmonary artery and veins to KCl and acetylcholine

**Fig. 5:**
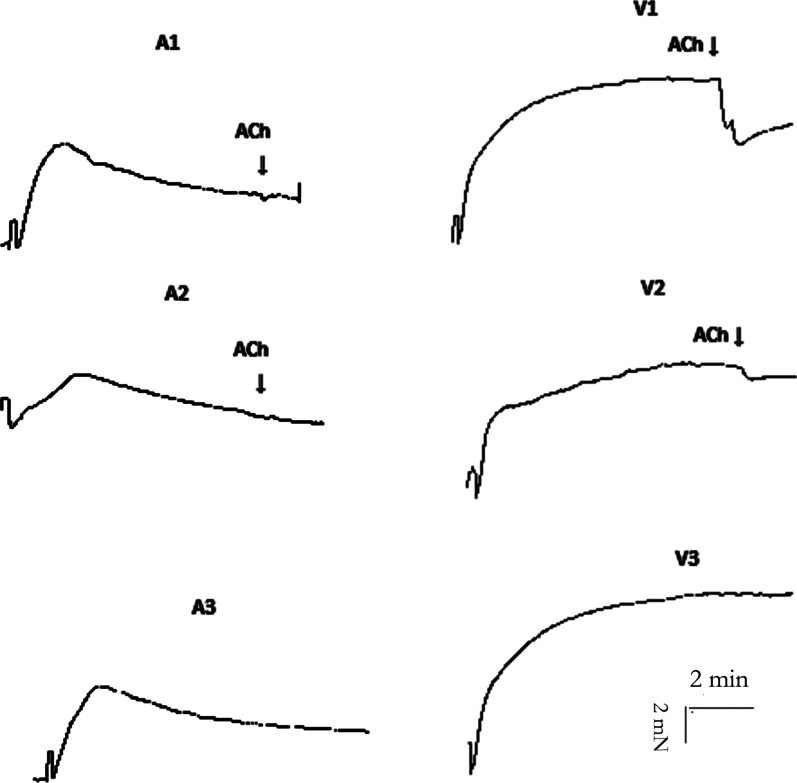
Responses of pulmonary arteries and veins to KCl and acetylcholine. Isometric tension recording of the effect of potassium chloride (KCl, 60 mM) on the pulmonary artery and vein segments from rats (endothelial integrity: N = 14; endothelium damage: N = 3; incubation with L-NAME: N = 5) (original trace). The number of rats is the number of repetitions of each group. A1–A3 represent the response to KCl stimulation of the pulmonary arteries with regard to endothelial integrity, endothelium damage and after incubation with L-NAME, respectively. V1–V3 represent the response to KCl stimulation of pulmonary veins with regard to endothelial integrity, endothelium damage and after incubation with L-NAME, respectively. After the contractions stabilized, the segments were exposed to a single high concentration of acetylcholine (ACh, 10^− 3^ M) (arrows)

After adding KCl, the vascular tension increased rapidly to a maximum value and then decreased slowly in pulmonary artery with an intact endothelium, endothelium-injured pulmonary artery, and pulmonary artery pre-incubated with L-NAME. Regardless of whether the vascular endothelium of the pulmonary artery was damaged, acetylcholine could not relax the arteries. After adding KCl, the vascular tension first increased rapidly and then increased slowly and finally stabilized at the maximum tension value in pulmonary veins with intact endothelium, damaged endothelium, and pulmonary veins incubated with L-NAME. Acetylcholine clearly relaxed the pulmonary vein with intact endothelium, and slightly relaxed the pulmonary vein with damaged endothelium. Response of pulmonary artery and pulmonary veins to KCl were independent of L-NAME incubation (Fig. 5).

### Response of pulmonary artery and pulmonary veins to acetylcholine

**Fig. 6:**
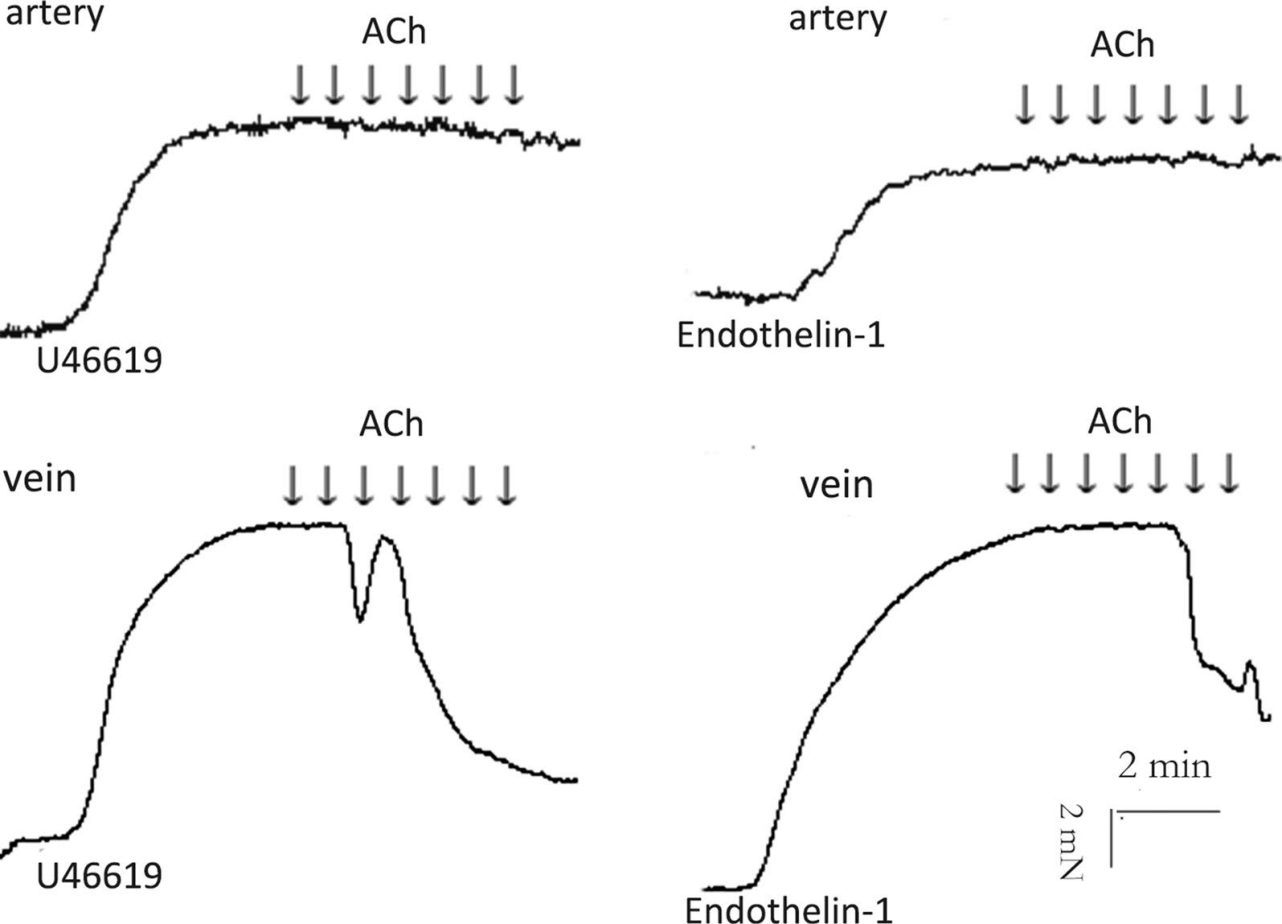
Responses of the pulmonary arteries and veins to acetylcholine. Isometric tension recording of the effect of acetylcholine (10^− 9^~10^− 3^ M) on pulmonary artery and vein segments pretreated with U46619 or endothelin-1 rats (N = 5). The number of rats is the number of repetitions of each group

Pulmonary artery pre-constricted with U46619 and endothelin-1 had no relaxation response to acetylcholine; whereas pulmonary veins pre-contracted with U46619 and endothelin-1 exhibited significant relaxation response to acetylcholine (Fig. 6).

## Discussion

The main findings of the in vitro study in healthy rats were as follows. First, phenylephrine exerted a relaxing effect on the pulmonary artery through activation β-receptor and increasing NO synthesis, but demonstrated expected constriction effect on pulmonary veins. Second, AVP relaxed pulmonary arteries significantly with or without L-NAME incubation. After incubation with L-NAME, AVP constricted the pulmonary veins more strongly. Third, the differences between pulmonary arteries and pulmonary veins was also reflected in the response to KCl and acetylcholine.

A recent study demonstrated that phenylephrine inhibits myometrium contraction in mice, with an IC_50_ of approximately 10^− 6^ M through activation of the β_2_-receptor in a concentration-dependent manner [8]. Our experimental findings were similar to this conclusion. Phenylephrine itself did not constrict pulmonary arteries, but produced significant constriction effect at concentration ≥ 10^− 6^ M after pretreatment with propranolol. Furthermore, after incubation with α1-blockers, phenylephrine significantly relaxed the pulmonary artery at concentrations ≥ 10^− 6^ M in a concentration-dependent manner, while the relaxation effect of phenylephrine decreased remarkably after further inhibiting β-receptor. At the same time, the rising speed of the constriction curve with L-NAME pretreatment slowed down and then declined at drug concentrations > 10^− 6^ M, which was also due to β-receptor activation. Therefore, constriction curves of the pulmonary arteries pretreated with both propranolol and L-NAME did not exhibit this phenomenon in the same phenylephrine concentration range. This suggested that phenylephrine was not a pure α1-receptor agonist as known currently because it also exerted a significant β-receptor agonist effect at concentrations > 10^− 6^ M.

We also found that after inhibiting NO synthetase phenylephrine concentration-dependently constricted the pulmonary artery even at low concentration. Additionally, in the relaxation experiment, phenylephrine still significantly relaxed the pulmonary artery at 10^− 6^ M after pretreatment with prazosin and propranolol, which was due to NO synthesis. We also noticed that at concentration > 10^− 6^ M the constriction effect of phenylephrine decayed on pulmonary artery pretreated with L-NAME. And the relaxation effect of phenylephrine caused by NO synthesis was only significant at 10^− 6^ M and reduced at concentrations > 10^− 6^ M. This suggested that the relaxation effect of phenylephrine caused by NO synthesis mainly occurred at concentrations ≤ 10^− 6^ M.

Collectively, there were three effects on pulmonary artery induced by phenylephrine: the constriction effect following α1-receptor activation; the relaxation effect after activation of the β-receptor; and the relaxation effect of NO synthesis. Ultimately, the effect of phenylephrine on pulmonary artery is relaxation. One supported study demonstrated that during exercise, when the levels of sympathetic active substances increase to activate lots of α and β receptors, as well as the synthesis of NO in large quantities, the balance is conducive to net mild pulmonary vasodilation [12]. Clinical studies also demonstrate that norepinephrine infusion increase mean arterial pressure while not affecting or decreasing pulmonary artery pressure [13, 14]. In summary, the relaxation effect of phenylephrine was mainly due to the production of NO at concentrations ≤ 10^− 6^ M while activating β-receptors at concentrations > 10^− 6^ M. That was also the reasons why the maximum pulmonary artery constriction effect induced by phenylephrine after pretreatment with propranolol and L-NAME was not significantly stronger than that of after propranolol or L-NAME pretreatment alone.

Our study also suggested that after pre-incubated with L-NAME and propranolol, although the constriction effect of pulmonary arteries stimulated by phenylephrine was significantly increased, it was still weak (17.2 ± 3.0 %). We speculated that endothelium dependent hyperpolarizing factor (EDHF), one component of endothelium-dependent relaxation, may also play an vital role in the tension of pulmonary arteries. The vasodilation mechanism of EDHF can be directly caused by calcium-activated potassium channels, or conducted by CX40 protein in gap junction after hyperpolarization of endothelial cells [15]. There are three subtypes of calcium-activated potassium channels related to the action of EDHF, namely large conductance calcium-activated potassium channel (BKCa), medium conductance calcium-activated potassium channel (IKCa) and small conductance calcium-activated potassium channel (SKCa). Garland et al. [16] found that BKCa was located on the smooth muscle membrane and was the binding site of EDHF; IKCa and SKCa only appear in endothelium cells [17]. Due to this distribution of calcium-activated potassium channels, one previous study have shown that the vasodilation of EDHF was attenuated by NO synthase inhibition in the pulmonary artery of rats [18], while another studies have reported that the vasodilation effect of EDHF continues significantly in the presence of the combination of L-NAME and the cyclooxygenase inhibitor indomethacin [19]. One recent study supported that after inhibition BKCa, phenylephrine-induced contraction of pulmonary artery rings enhanced about 25% [20].

However, phenylephrine concentration-dependently constricted pulmonary veins, and there was no difference in the contractile effect of phenylephrine in each group with or without pre-incubation with propranolol or/and L-NAME. We concluded that for pulmonary veins, phenylephrine demonstrated only a contractile effect through activating ɑ1 receptor with little NO production or β-adrenergic receptor activation effect.

We speculated that the difference between the pulmonary arteries and veins was due to different distribution of β-receptors and production of NO. Compared to pulmonary vein, pulmonary artery had a denser distribution of β-receptors and a greater amount of NO production. An earlier study also supported this conclusion, suggesting that most of the effects of beta blockade and EDRF suppression occur mainly in pulmonary artery and extra-alveolar blood vessels [12]. We believed that the relaxation response of pulmonary artery to the adrenergic receptor agonist was a self-protection mechanism of the healthy organism to avoid pulmonary arterial hypertension while increasing mean arterial pressure.

While investigating the different responses of the pulmonary artery and veins to AVP, we found that AVP significantly relaxed pulmonary artery pre-constricted with U46619, and achieved the maximum relaxation effect at a concentration of 3 × 10^− 8^ M. Other studies involving rats also demonstrated that AVP reduced the pulmonary vascular resistance index in vivo [21] and in vitro (6). Studies also showed that vasopressin-induced pulmonary artery relaxation was caused by the production of NO [22, 23] mediated by V1-receptor in the intact vascular endothelium [23]. However, our experiment also revealed that AVP still significantly relaxed pulmonary artery after pre-incubation with the NO synthase inhibitor L-NAME, which although was obviously weaker than that in untreatment group. Moreover AVP still hardly constricted L-NAME-pretreated pulmonary artery. Therefore, we believed that AVP relaxed pulmonary artery not only through NO synthesis pathway(s). A previous study reported that AVP reversed approximately 65 % of U-46,619-induced pulmonary vascular constriction, mainly caused by the activation of V1 receptors; AVP also produced approximately 8 % pulmonary artery relaxation by activating V2 receptors [24]. We speculate that this non-NO synthesis pathway was mediated by V2 receptor. AVP constricted pulmonary veins more significantly after L-NAME pre-incubation, which indicated AVP could induce NO production in the pulmonary veins.

Our study also found that differences in changes in vascular tension stimulated by KCl between pulmonary arteries and veins was also one of the methods to distinguish the two in vitro. It was not related to the integrity of the vascular endothelium or the amount of NO production. We speculated that this was probably due to the different arrangement of smooth muscle layers in pulmonary artery and veins and the different characteristics of ion channels in smooth muscle. BKCa is the predominant potassium channel of vascular smooth muscle, and its activation negatively regulates vascular tone. The cell membrane depolarization increases vascular tension through increasing intracellular calcium concentration; and the increasing Ca2 + activates calcium-activated potassium channels results potassium outflow, which leads to cell membrane potential repolarization and superpolarization; and following voltage-dependent Ca2 + influx decreases or stops, the tension of vascular smooth muscle gradually decreases [25–27]. In KCl stimulation experiment, the phenomenon that pulmonary artery tension gradually decreased after rapidly reaching maximum value was probably caused by BKCa. Another supporting study also showed that EDHF-mediated relaxation was sensitive to 60mM K(+) depolarizing solution in pulmonary arterial rings [18]. The calcium-activated potassium channels seen in smooth muscle cells also change from BKca to IKca predominance in PAH [28]. One literature also suggested that the relaxation of EDHF is influenced by multiple factors, including vascular distribution and caliber, pre-existing tone [29]. This suggested that the BKCa activity and quantity in the pulmonary vein smooth muscle may be significantly different from that of pulmonary artery, which leaded to different constriction curves in potassium stimulation.

We also found that the endothelial function of pulmonary veins could be determined by the relaxation effect of acetylcholine. However, pulmonary arteries could not be relaxed by acetylcholine regardless of whether the endothelial of vessels were damaged. Pulmonary artery pre-constricted by U46619 and endothelin-1 still cannot be relaxed by acetylcholine while acetylcholine clearly relaxed such pre-constricted pulmonary veins. Although previous studies and ours have confirmed that AVP can significantly relax the pulmonary artery, but our experiments also revealed that this relaxation effect also occurred after the inhibition of NO synthase. Therefore, the relaxation effects of AVP do not appear to be a good predictor of endothelial function in pulmonary arteries.

Earlier in vitro study showed that phenylephrine could not cause the constriction of pulmonary veins in either chronic hypoxic or healthy rats, but could concentration-dependently constricted pulmonary artery; moreover, acetylcholine clearly relaxed U46619-precontracted pulmonary artery, but hardly relaxed pulmonary vein [7]. These results contradicted those of ours. The methods of free pulmonary artery and veins may further support the reliability of our study. We adopted a method of gradually approaching the target blood vessels from the outside to the inside. And the target blood vessel was always connected to the pulmonary vascular trunk during separation lung tissue to ensure the accuracy of the pulmonary arteries or pulmonary veins.

The tracheal tissues at all levels are closely intertwined with the pulmonary artery and pulmonary veins to maintain high-efficiency gas exchange. There are also bronchial circulation vessels with a nutrient function that belong to the systemic circulatory system. The further these tissues are from the hilum of the lung, the more difficult it is to distinguish them, especially after separation from the origin of the lumen tissue. Our study demonstrated that pulmonary artery and pulmonary veins could be distinguished by different responses to common drugs (i.e., KCl, phenylephrine, and acetylcholine), which improves the efficiency and accuracy of relevant in vitro experiments.

Nevertheless, there were still some limitations in the present study. We did not explore whether phenylephrine also exerts such different effects in pulmonary vascular with diseases. We did not further explore which subtype of beta receptor was activated by phenylephrine. Differences in the distribution of adrenergic receptors and the expression of NO synthase between the pulmonary artery and veins were not explored at the molecular level. Some studies have shown that phenylephrine significantly constricts the pulmonary arteries of humans, dogs, pigs, and cattle [10, 23, 30, 31]; therefore, we speculate that the effect of phenylephrine on the pulmonary vascular may have significant species differences.

## Conclusions

Our study demonstrated that phenylephrine significantly relaxed the pulmonary artery through the activation of β-adrenoreceptors and inducing NO production, but clearly constricted the pulmonary veins. We believe that AVP relaxed the pulmonary artery by producing NO, as well as non-NO synthesis pathways. When exposure to KCl, pulmonary artery tension gradually decreased after rapidly reaching maximum value, while pulmonary vein rapidly reached the maximum tension and stabilized at the maximum value. Different from the phenomenon presented in veins, acetylcholine did not relax pulmonary artery preconstricted by KCl, U46619, and endothelin-1. Obtaining human pulmonary vessels is difficult due to issues such as lack of access and ethics. Therefore, it is very popular to use rodents to study the occurrence and development of lung diseases. In vitro, the intralobar branches of pulmonary artery and pulmonary vein are easily confused due to similar small lumens, adjacent locations. According to our results, commonly used vasoactive drugs could quickly and effectively distinguish pulmonary arteries and pulmonary veins, which improves the accuracy and effectiveness of the relative experiment.

## Data Availability

Data are available from the authors upon reasonable request.

## Abbreviations

ACh: Acetylcholine
NO: Nitric oxide
EDRF: Endothelium-derived relaxing factor
AVP: Arginine vasopressin
KCl: Potassium chloride
L-NAME: N[gamma]-nitro-L-arginine methyl ester
EDHF: Endothelium derived hyperpolarizing factor
